# Editorial: Future directions in conduction system pacing to achieve cardiac resynchronization

**DOI:** 10.3389/fphys.2023.1281552

**Published:** 2023-09-13

**Authors:** Vishal S. Mehta, Steven Niederer, Christopher A. Rinaldi

**Affiliations:** ^1^ Cardiology Department, Guy’s and St Thomas’ NHS Foundation Trust, London, United Kingdom; ^2^ School of Biomedical Engineering and Imaging Sciences, King’s College London, London, United Kingdom; ^3^ Biomedical Engineering, National Heart and Lung Institute, Imperial College London, London, United Kingdom

**Keywords:** cardiac resynchronization therapy, physiological pacing, conduction system pacing, CSP, CRT

The first clinical use of cardiac resynchronization therapy (CRT) delivered by biventricular pacing (BVP) was almost 30 years ago (Cazeau et al., 1994). Since then, CRT has revolutionized the management of patients with heart failure and dyssynchronous ventricular contraction, for which the next step was previously heart transplantation. Over this period, multiple randomized studies involving thousands of patients, including MADIT-CRT, COMPANION, and MIRACLE ICD (Bristow et al., 2000; Young et al., 2003; Zareba et al., 2011), have consistently shown the benefit of biventricular CRT in terms of morbidity and mortality in heart failure patients with left bundle branch block (LBBB). The use of BVP has been exhaustively explored in different clinical scenarios. However, the non-response rate remains fiendishly stubborn at around 30% (Daubert et al., 2017). “Deus ex machina” describes a situation in which a seemingly unsolvable problem is abruptly resolved by an unexpected occurrence. Is conduction system pacing such a remedy for those left behind in BVP’s journey?

BVP is non-physiological and only modestly reduces ventricular resynchronization through the fusion of two wave fronts from the LV epicardium and RV endocardium. In comparison, CSP delivered as His bundle pacing (HBP) or left bundle branch area pacing (LBBAP), can mimic the physiological form of electromechanical coordination. Theoretically, HBP would be most physiological by simultaneously activating both ventricles, whereas LBBAP should capture the LBB, thereby reducing QRS duration and restoring LV activation (Curila et al., 2021). Clinical trial evidence for CSP remains limited in comparison with the vast trial data supporting BVP. Only four randomized controlled trials (RCTs) have been performed to date involving CSP: His-Sync (41 patients), His-Alternative (50 patients), LEVEL-AT (70 patients), and LBBP-RESYNC (40 patients) (Upadhyay et al., 2019; Michael et al., 2021; Margarida et al., 2022; Yao et al., 2022). These are small numbers compared with MADIT CRT, which enrolled 1,820 patients alone. Nevertheless, pooled meta-analyses, including both randomized and non-randomized studies comparing CSP with BVP, have been undertaken. Of the 15 studies included in Zhang et al.’s meta-analysis, only four were randomized and the results suggested CSP is associated with shortened QRS duration and an improved LVEF and NYHA class. Pooling study designs in such a way is fraught with challenges in interpretation and these findings suggest cautious optimism.

In this Research Topic, we overview CSP from an electromechanical perspective, its role in specific patient groups, and perspectives on lead management and developments in delivering CSP. Bressi et al.’s detailed review acknowledges the role of CRT delivered by BVP. However, the author also correctly identifies its shortcomings depending on coronary venous anatomy and its non-physiological activation patterns. The Mini Review highlights the results of observation studies showing a 90% success rate of HBP. However, small RCTs in the field showed high crossover rates of up to 48% in HBP groups, which is higher than BVP failure (Upadhyay et al., 2019). In comparison, the relatively high success rate of LBBAP ranging from 80% to 97% is encouraging, alongside observational studies showing better clinical outcomes compared with BVP in patients with LBBB.

Kong et al.’s review of pacing considerations in LBBB covers the heterogeneous nature of this conduction pattern. It expertly explains how LBBAP should be considered in a complete conduction block and how hybrid models using His-optimized CRT (HOT-CRT), or left bundle branch optimized CRT (LOT-CRT), may be more appropriate for those with interventricular conduction delay with preserved His-Purkinje activation (IVCD with IPA) or a combination of both proximal block and concomitant distal disease.

Strocchi et al.’s modeling study shows that patients with RBBB significantly benefit from HBP but not BVP CRT or left bundle pacing, and that the location at which conduction velocity is slowest—whether or not the myocardium is healthy—has a significant impact on the effectiveness of the different pacing locations. Further simulation work by the same group has also shown how septal scar is a significant factor making CSP ineffective (Strocchi et al., 2023). Again, patient selection is clearly of the utmost importance when considering the type of CSP—a similarity to the challenges facing BVP-delivered CRT.

Chubb et al., reviews the potential role of CSP in a pediatric population and patients with adult congenital heart disease (ACHD). CSP in this population has huge potential advantages, particularly for the chronically paced pediatric population. Nevertheless, this must be balanced against the increased procedural complexity and procedural risk in this patient group. The author anticipates that CSP in this population cohort will continue to grow.

As the number of implanted CSP leads increases, a significant minority of these patients will require revisions, and their leads may become infected and require extraction. Wijesuriya et al. review the role of transvenous lead extraction (TLE) in CSP. A retrospective analysis of 30 patients with HBP leads requiring TLE showed that 22 were due to high thresholds (Vijayaraman et al., 2019). With respect to TLE in left bundle pacing leads, this Mini Review has only identified three case reports, all of them involving leads *in situ* for <2 years. As left bundle pacing requires screwing the lead relatively deeply into the septum, concerns remain regarding iatrogenic septal damage from lead extraction. The removal of leads is an important safety factor to consider when deciding on implantation—only time will clarify the ease of extraction and its long-term sequelae. A final Frontier may well be the role of leadless CSP. Finally, the authors provide a detailed hypothesis of how leadless pacing via the Wise-CRT system could be deployed and examine the potential benefits of this. This remains in its infancy, and further studies will need to be conducted on this novel technology.

A recent survey of European electrophysiologists showed the expectation that some form of LBBAP or HBP will dominate future bradycardic (85%) and CRT implants (72%) (Kircanski et al., 2023). At present, guidelines do not reflect this (Glikson et al., 2021), but there is no doubt that change is on the horizon. While the temptation to implant CSP leads is strong, there is currently a lack of randomized trial data to support the routine use of CSP in patients who are currently indicated for CRT, and operator experience will be important for producing reproducible outcomes. CSP may well represent the “deux ex machina” for the challenges faced in cardiac resynchronization delivery; nonetheless, a healthy dose of patience and caution is advised.
